# Influence of upflow velocity on performance and biofilm characteristics of Anaerobic Fluidized Bed Reactor (AFBR) in treating high-strength wastewater

**DOI:** 10.1186/s40201-014-0139-x

**Published:** 2014-11-25

**Authors:** Jalil Jaafari, Alireza Mesdaghinia, Ramin Nabizadeh, Mohammad Hoseini, Hossein kamani, Amir Hossein Mahvi

**Affiliations:** School of Public Health, Tehran University of Medical Sciences, Tehran, Iran; Department of Environmental Health Engineering, School of Public Health, Shiraz University of Medical Sciences, Shiraz, Iran; Health Promotion Research Center, Zahedan University of Medical Sciences, Zahedan, Iran; Center for Solid Waste Research, Institute for Environmental Research, Tehran University of Medical Sciences, Tehran, Iran; National Institute of Health Research, Tehran University of Medical Sciences, Tehran, Iran

**Keywords:** Biofilm characteristics, Biomass concentration, Anaerobic Fluidized Bed Reactor, Currant wastewater, Upflow velocity

## Abstract

One of the key parameters in Fluidized Bed reactors is the control of biofilm thickness and configuration. The effect of upflow velocity on performance and biofilm characteristics of an Anaerobic Fluidized Bed Reactor was studied in treating Currant wastewater at various loading rates. The reactor used this study was made of a plexiglass column being 60 mm diameter, 140 cm height, and a volume of 3.95 L. The results demonstrated that the AFBR system is capable of handling an exceptionally high organic loading rate. At organic loading rates of 9.4 to 24.2 (kg COD m^−3^) at steady state, reactor performances with upflow velocities of 0.5, 0.75 and 1 (m min^−1^) were 89.3- 63.4, 96.9 – 79.6 and 95 – 73.4 percent, respectively. The average biomass concentration per unit volume of the AFBR (as gVSSatt L^−1^ expended bed) decreased with the increase of upflow velocity in the range of 0.5–1 m min^−1^ at all applied organic loading rates. The total biomass in the reactor increased with increases in the organic loading rate. The peak biomass concentration per unit volume (as gVSSatt L^−1^ expended bed) was observed at the bottom part of the reactor, then it droped off slowly towards the top. The biofilm thickness increased from the bottom to the top of the reactor representing a stratification of the media in the AFBR. The bed porosity increased from the bottom to the top of the reactor.

## Introduction

In recent years many alternatives have been performed to treatment of high-strength wastewaters [1–4]. Anaerobic Fluidized Bed Reactors (AFBR) were originally a chemical engineering tool used to perform phase transformations, reactions, and diffusions of various chemicals existing in solid, liquid, and vapor phases. With the concept of maximum diffusion and maximum chemical reaction within a minimum volume in mind, AFBRs have been used in biological wastewater treatment and are utilized in several process configurations [5–7]. The results from recent studies have consistently illustrated the technical advantage of the fluidized bed over most other suspended and attached growth biological systems. Typically, in a similar capacity, efficiency of the AFBR can be more than 10 times of the activated sludge system while the total space occupied by AFBR is about 10 percent of the required space for stirred tank in activated sludge process [8]. This is due to the AFBR ability in maintaining high concentration of biomass compared with conventional activated sludge system (40,000 mg L^−1^ vs. 3000 mg L^−1^) [9]. Fluidization can overcome operating problems such as bed clogging and high pressure drop, which happen if the media with high surface area used in packed-bed reactor. Another advantage of using media is possibility of elimination of secondary clarifiers [10]. Anaerobic Fluidized bed reactors (AFBR) are high-load wastewater treatment systems, which have been studied by numerous authors to treat different industrial wastewaters. For example, this system has been used for treatment of textile wastewater [11], ice-cream wastewater [12], and brewery wastewater [13], winery wastewater from Grape-Red and tropical fruit [14], currant [15] and sanitary landfill leachate [16]. The microbial population is the critical parameter in the performance of biological process that the influenced by operational parameters, physicochemical properties of the carrier material (density, roughness, porosity) on the fixed bed process are critical considerations [17,18]. One of the key operational parameters in attached biofilm reactors is the control of biofilm thickness and configuration, and research on biofilm formation and detachment has developed considerably in the past years, although there is no design rule for the rate of detachment. The prediction of biofilm structure (density, porosity, roughness, shape) and thickness is most important in designing and operation of biofilm processes, because hydrodynamics, mass transfer and conversion in biofilm processes depend on these variables. In attached growth process, biofilm accumulation is a dynamic process that is the net result of growth and the detachment processes. This is affected by several external factors, including composition and concentration of the feed, concentration of particles, particle–particle collisions, and particle–wall collisions, and velocity of the liquid phase (shear stress). This is the most important factor influencing formation, structure and stability of biofilms. In a biofilm system, higher hydrodynamic shear force take a stronger biofilm, and the biofilm tends to become a heterogeneous, porous and weaker structure when the shear force is too weak [19–22].

The main objective of this study was to investigate the influence of different upflow velocity on performance and biofilm characteristics of Anaerobic Fluidized Bed Reactor in treating a real Currant wastewater in various HRT and loading rates.

## Materials and methods

### Anaerobic Fluidized bed reactor

**Figure 1:**
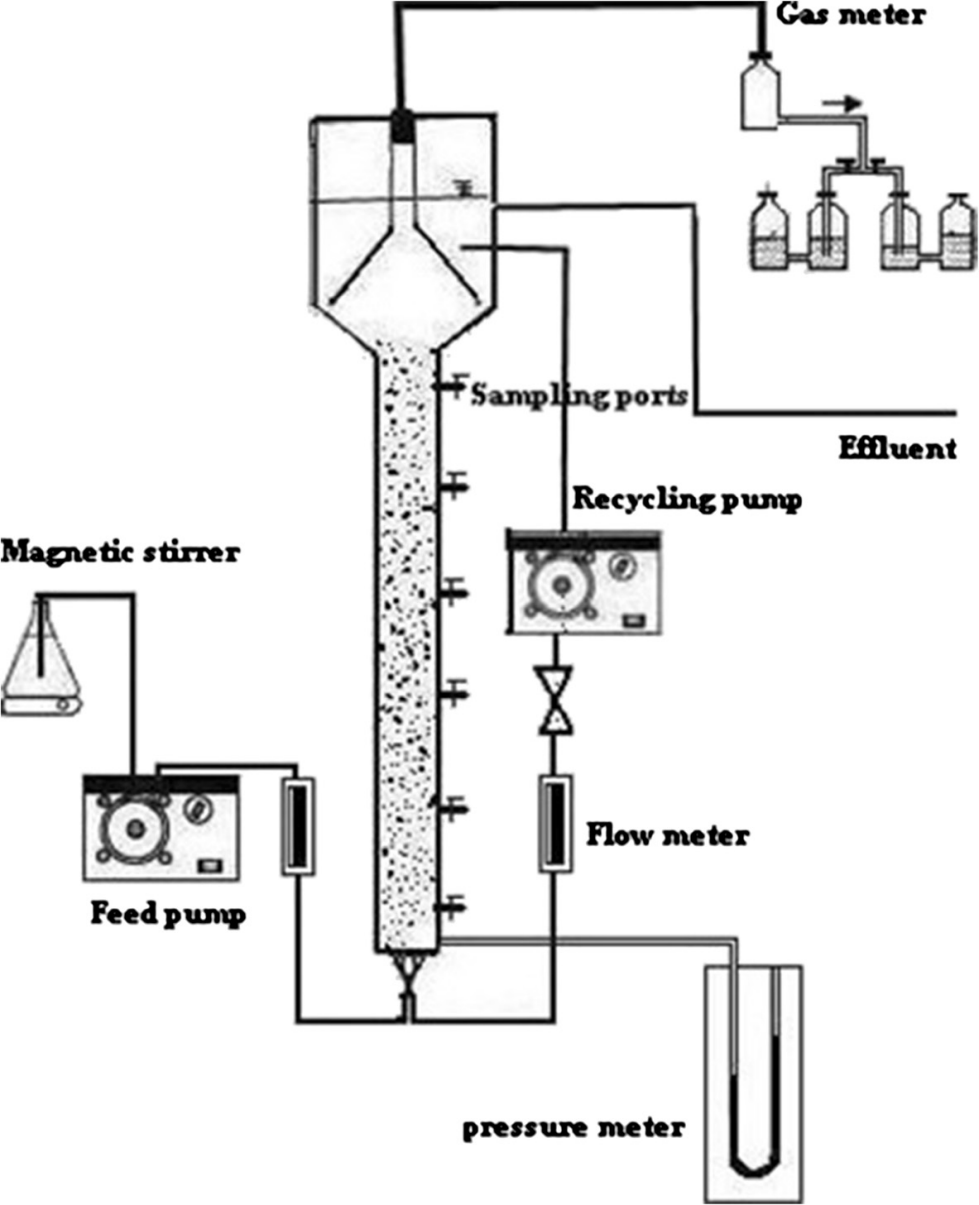
**Schematic configuration of Anaerobic Fluidized bed reactor.**

**Figure 2:**
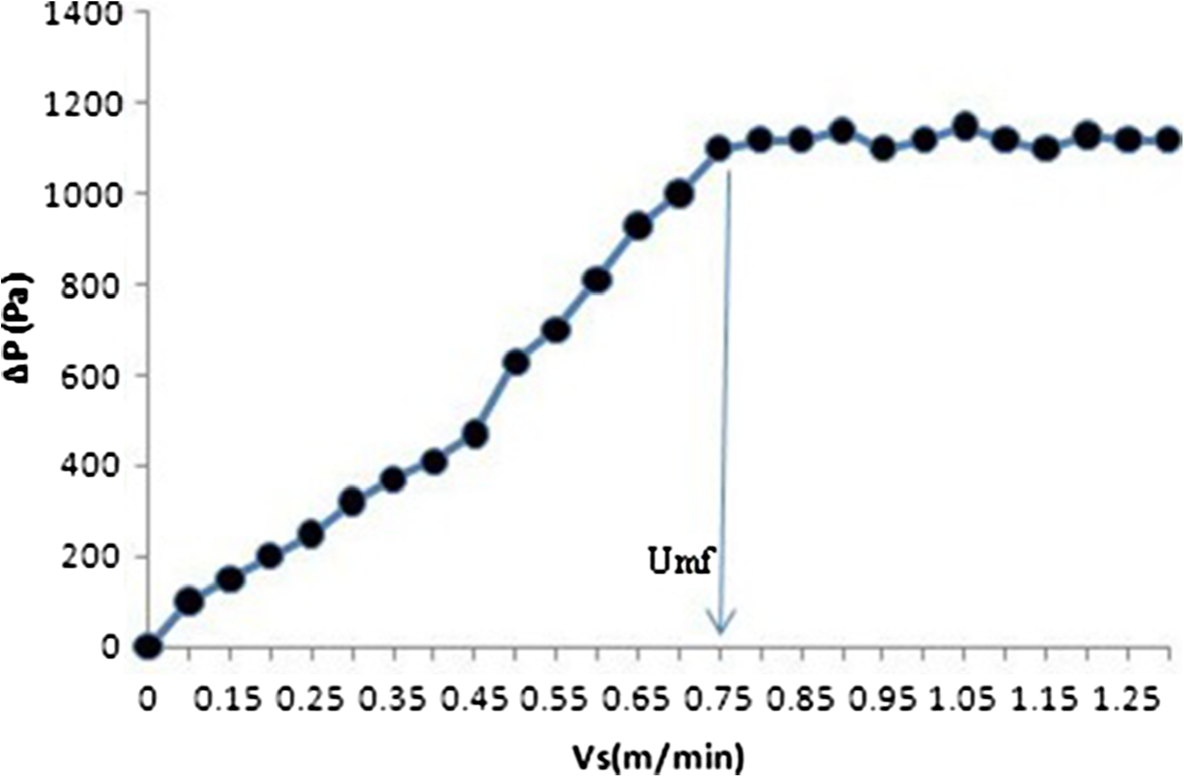
**Effect of liquid upflow velocity on pressure drop.**

The reactor was made of a plexiglass column being 60 mm diameter, 140 cm height, and a volume of 3.95 L. The enlarged top section of column was used as a gas–solid separator. The enlarged section had diameter of 100 mm with a volume of 1.48 L (Figure 1). The bottom of the reactor was flat with symmetrically placed four pores through which flow was equally distributed into the reactor. The column has six sampling ports located at 5, 30, 55, 80, 105 and 130 cm above the reactor bottom. The recycled flow was drawn from the top section using a Circulator Pump and then fed upward into the reactor. Reactor temperature was controlled by Aquarium Heater at 35 ± 2°C. The reactor was loaded with 1.48 kg media made of PVC with a mean diameter of 2 mm as a biofilm carrier to a settled depth of 0.6 m. The particles had a specific gravity of 1.45, a porosity of 0.4, and a specific surface area of 1800 m^2^ m^−3^. The bed expansion in fluidized bed reactor was 30% during the start-up period. The expansion of the bed should be determined based on consideration of the minimum fluidization velocity. Some studies have been determined the factors affecting minimum fluidization velocity and maximum pressure drop [23–25]. Also, Peng and Fan [26] developed theoretical models for estimating of minimum fluidization velocity and maximum pressure drop, based on the dynamic balance of forces exerted on the particle. Some of the well known correlations available for predicting the minimum fluidization velocity (*U*mf) and maximum pressure drop (*P*max) for tapered beds are those by Peng and Fan. The influence of superficial velocity on pressure drop in reactor is illustrated and shown in Figure 2. By increasing upflow velocity to 0.75 (m min^−1^), pressure drop increased, then, with increasing superficial velocity to more than 0.75 (m min^−1^), pressure drop remained constant. So, to ensure that the fluidization condition is exist, the superficial velocity should not be less than the minimum fluidization velocity which is 0.75 m min^−1^ in our reactor. Also, upflow velocity in start-up period was adjusted 0.75 (m min^−1^). After the start-up period, the real Currant wastewater was fed to the reactor and upflow velocities in different organic loading rate were adjusted to 0.5, 0.75 and 1 m min^−1^.

### Start-up period

Anaerobic reactor was seeded with 1 L of aerobic active sludge obtained from aerobic digesters of municipal wastewater treatment plant with MLSS and MLVSS of 24.84 and 16.9 g L^−1^, respectively. Table 1 is a summary of conditions tested during the start-up. The reactor fed with synthetic wastewater contained methanol, glucose, and Currant wastewater. Some macro and micronutrients such as CaCl_2_.2H_2_O (50 mg L^−1^), (NH_4_)_2_.HPO_4_ (80 mg L^−1^), FeCl_2_.4H_2_O (40 mg L^−1^), NH_4_Cl (1200 mg L^−1^), Na_2_S.9H_2_O (300 mg L^−1^), CuCl_2_.2H_2_O (0.5 mg L^−1^), MgSO_4_.7H_2_O (400 mg L^−1^), H_3_BO_3_ (0.5 mg L^−1^), MnCl_2_.4H_2_O (0.5 mg L^−1^), NaWO_4_.2H_2_O (0.5 mg L^−1^), AlCl_3_.6H_2_O (0.5 mg L^−1^), Na_2_SeO_3_ (0.5 mg L^−1^), mg/l), KCl (400 mg L^−1^), ZnCl_2_ (0.5 mg L^−1^), NaHCO_3_ (3000 mg L^−1^), NaMoO_4_.2H_2_O (0.5 mg L^−1^), CoCl_2_.6H_2_O (10 mg L^−1^), KI (10 mg L^−1^), and NiCl_2_.6H_2_O (0.5 mg L^−1^), which are needed for optimal biofilm growth were used. In colonization stage, anaerobic fluidized reactor was run in the batch mode for one week. Then, during the start-up period run in the continuous mode and COD concentration in feed was gradually raised. Also methanol, which included 75% of the total influent COD, was used in the beginning to encouraging the growth of methanosarcina bacteria [27]. Then, the percent of methanol in the influent was gradually decreased to 50%, 25%, and 0% in days 11, 21, and 31, respectively by replacing with glucose and Currant wastewater. Additionally, in the start-up period, NH_4_Cl concentration was gradually increased to taken high C/N ratios (1200 mg L-1). Part of this N with carbon was used by bacteria for building up the new cell to encourage extra cellular polymer production, which aids bacterial attachment on solid surface [28].

**Table 1:** **Organic loading and characteristics of fed during the start-up**

**Time (d)**	**COD loading (kg COD/m** ^**3**^ **)**	**Methanol a**	**Glucose a**	**Currant wastewater a**	**NH**_**4**_ **Cl b**
0-10	0.5 - 4	75	25	0	50
11-20	4 - 7	50	50	0	75
21-30	7 - 11	25	75	0	100
31-40	11 - 13	0	75	25	100
41-50	13 - 15	0	50	50	100
51-60	13 - 15	0	25	75	100

a- % of total COD b- % of its value at the end of the start -up.

### Operation period

In the operational period that lasted 372 d, the Anaerobic Fluidized bed reactor was fed with real Currant wastewater. The real currant wastewater obtained from the factory located in the Safadasht Industrial Zone, Shahriar, Iran, that in Characteristics of Currant wastewater is given in Table 2. The AFBR was operated under five different hydraulic retention times of 48, 40, 32, 24 and 18 h, respectively and each of HRT operated under three upflow velocities of 0.5, 0.75 and 1 m min^−1^, respectively.

**Table 2:** **Characteristics of currant wastewater used in the present study**

**Parameter**	**Value**		
	**Range**	**Average**	**SD**
pH Value	5.2-7.3	6	0.7
COD (mg/L)	17200-19000	18250	447
BOD5 (mg/L)	12500-13000	12748	185
TSS (mg/L)	331-410	365	23.3
COD–BOD ratio	1.45	-	-
Tot-P (mg/L)	12-25	18	3.8
Tot-N (mg/l)	41-86	60	13.1

### Analytical methods

Samples were analyzed for COD according to standard method [29]. Temperature was measured by a thermometer and pH was measured by a pH-meter (E520 Metrohm Herisau). The biofilm thickness was measured using the method of Schreyer and Coughlin [30], according to the following method. A slurry sample of known volume was smoothly washed to remove the suspended solids and then filtered. The wet bio-particles were carefully removed from the filter into a ceramic dish and weighed to determine its wet mass. After oven-drying for 24 h at 105°C, then cooled in a desiccator and weighed. The dried sample was ignited in a 550°C furnace for 30 min, cooled in a desiccator and then weighed. The difference between two dried weights would yield the weight of immobilized biomass as attached volatile solids (AVS). Also for ensure the results obtained from the Schreyer and Coughlin procedure, the biofilm thickness was measured using a high-resolution microscope equipped with a micrometer [30] method. In comparison of two measurements, the relative error was always less than 10%. The bio-particle density was measured from its settling velocity and diameter of bio-particle [31].

## Results and discussion

The start-up period was completed in 60 d. So, that the feed COD increased stepwise and effluent COD of the anaerobic decreased and the COD removal efficiency gradually increased. In the end of the start-up period, in Anaerobic Fluidized Bed Reactor, attached volatile solid (AVS) concentration reached to 0.0185 gvss g^−1^ which is in accordance with ranges 0.074–0.11 reported by Farhan et al., 1997 [32], 0.039 [33], 0.05 [34], 0.0732 [27] and 0.0375–0.429 gvss g^−1^ by [35]. Table 3 shows operational parameters obtained at the end of start-up period.

**Table 3:** **Operational parameters obtained at the end of start-up period for AFBR**

**Operational parameters**	**FBR**
OLR, kg COD/m3	15
HRT	24
Upflow velocity (m/min)CBU	0.75
Expansion %	30
Volume of expanded bed (cm3)	2210
M support (g)	1480
VSatt (g)	27.5
g VSatt/g support	0.0185
g VSatt/l expanded bed	11.9

### Effect of organic loading rate and upflow velocity on COD removal

**Figure 3:**
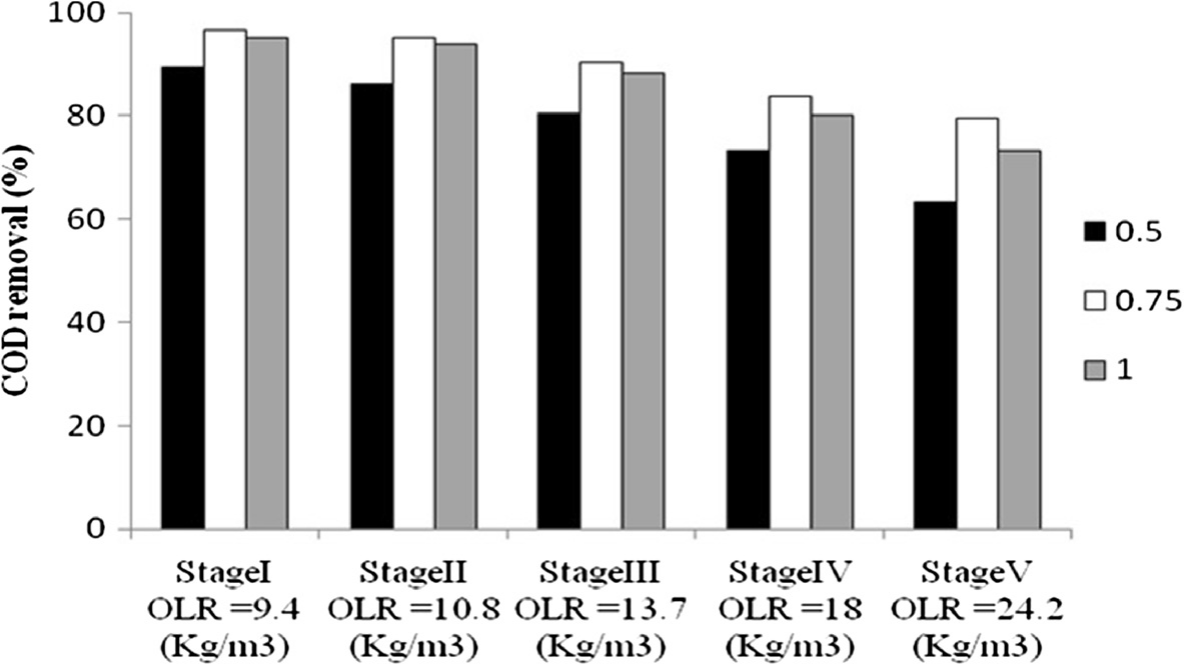
**Effect of the upflow velocity and organic loading rate on reactor performance.**

Figure 3 and Table 4 show the effect of the OLR on the COD removal efficiency (E) and COD effluent in reactor throughout the operation time for the reactor studied. As shown, during stage 1, OLR in AFBR was kept at around 9.4 g COD L.d^−1^ with the feed COD concentration of 18,000 ± 300 mg L^−1^ and HRT around 48 h, the reactor performance was investigate for 0.5, 0.75 and 1 m min^−1^ upflow velocities. At steady state, with 0.5, 0.75 and 1 m min^−1^ upflow velocities, the reactor performances in stage 1 were 89.3, 96.6 and 95 percent, respectively. At stage 2, the HRT of reactor was decreased from 48 h to 40 h and OLR in Anaerobic Fluidized Bed Reactor increased from 9.4 to 10.8 g COD L.d^−1^ and feed COD concentration was as same as the stage 1, the reactor performance dropped to 86, 95.2 and 94 percent, respectively. As can be seen, in the stage 1 and 2, in the second set, 0.75 m min^−1^ upflow velocity had more removal efficiency than other upflow velocities. Also In other stages, in the second set, 0.75 m min^−1^ upflow velocity had more removal efficiency than other upflow velocities. In the stage 5, the OLR was further increased to 24.2 g COD L.d^−1^. By decreasing HRT to 18 h, reactor performance dropped to 63.4, 79.6 and 73.4 percent, respectively. The average COD concentration in the effluent of the three sets at a loading rate of 9.4 g COD L.d^−1^ was 2020, 630 and 940 mg L^−1^, respectively. Then, the average COD concentration in the effluent of the three sets at a loading rate of 24.2 g COD L.d^−1^ increased gradually to 6515, 3666 and 4835 mg L^−1^, respectively.

**Table 4:** **Summary of the average results of the three sets of experiments at steady state**

**Stage**	**Time (d)**	**Vs (m/min)**	**Q**_**in**_ **(l/d)**	**HRT (h)**	**OLR (gCOD/l/d)**	**CODout (mg/l)**	**VS**_**att**_ **(gvs/l)**	**Expanded bed (mm)**
I	1-18	0.5	2.25	48	9.4 ± 0.2	2020	20.2	690
	19-37	0.75	2.25	48	9.4 ± 0.2	630	15.5	790
	38-61	1	2.25	48	9.4 ± 0.2	940	12.1	905
II	62-78	0.5	2.7	40	10.8 ± 0.2	1540	18.1	780
	79-97	0.75	2.7	40	10.8 ± 0.2	873	13.9	885
	98-118	1	2.7	40	10.8 ± 0.2	1086	11.7	945
III	119-138	0.5	3.375	32	13.7 ± 0.3	3440	17.1	835
	139-163	0.75	3.375	32	13.7 ± 0.3	1544	13.4	930
	164-188	1	3.375	32	13.7 ± 0.3	1780	11.2	1000
IV	189-210	0.5	4.5	24	18 ± 0.3	4815	16.8	870
	211-238	0.75	4.5	24	18 ± 0.3	2970	13.2	955
	239-267	1	4.5	24	18 ± 0.3	3650	10.8	1120
V	268-294	0.5	5.6	18	24.2 ± 0.5	6515	16.7	905
	295-326	0.75	5.6	18	24.2 ± 0.5	3666	13.18	980
	327-372	1	5.6	18	24.2 ± 0.5	4835	10.8	1180

Higher biodegrading rates were generally achieved at relatively lower superficial velocities. However there was a minimum practical velocity (0.5 m min^−1^) below which would agglomeration of media occur in the reactor and the anaerobic process might disrupt. Also the subsequent decrease of the fluidization percentage in 0.5 m min^−1^ upflow velocity, which is below the minimum fluidization velocity, might have mass transfer limitations caused by accumulation of fatty acids in the reactor [36]. The substrate utilization rate in the biological process, correlated to diffusion resistance, is strongly dependent on reactor design and mixing intensity [37]. In the third set in Vs of 1 m min^−1^, the reactor performance was lower in compare with the second set with the Vs of 0.75 m min^−1^, because the biofilm was detached and washed out of the system as a result of the increased shearing force and bed porosity. In the treatment of high-strength distillery wastewater by anaerobic fluidized bed reactor with natural zeolite, COD removals of 80% were achieved at OLR of 20 g COD L.d-1 and HRT of 11 h [38]. In another study with anaerobic fluidized bed reactor for treating ice-cream wastewater, at an organic COD loading rate of 15.6 g L-1. d and HRT of 8 h, COD removal efficiencies of 94.4% was achieved [12]. In the treatment of thin stillage wastewater using an anaerobic fluidized bed with OLR of 29 g COD L.d-1 and HRT of 3.5 h, COD removal efficiencies of 88% was achieved [38]. In the stage 1 and 2, with increasing the upflow velocity, COD removal rate due to appropriate mass balance was improved. But, in the stages 3 to 5, in three set with 0.75 m min^−1^ upflow velocity, Vs was increased due to the decrease in the biomass concentration, which resulted increase in shearing force and increase in bed porosity, while the organic loading rate in the reactor was increasing.

### Effect of the upflow velocity and organic loading rate on the biomass concentration

**Figure 4:**
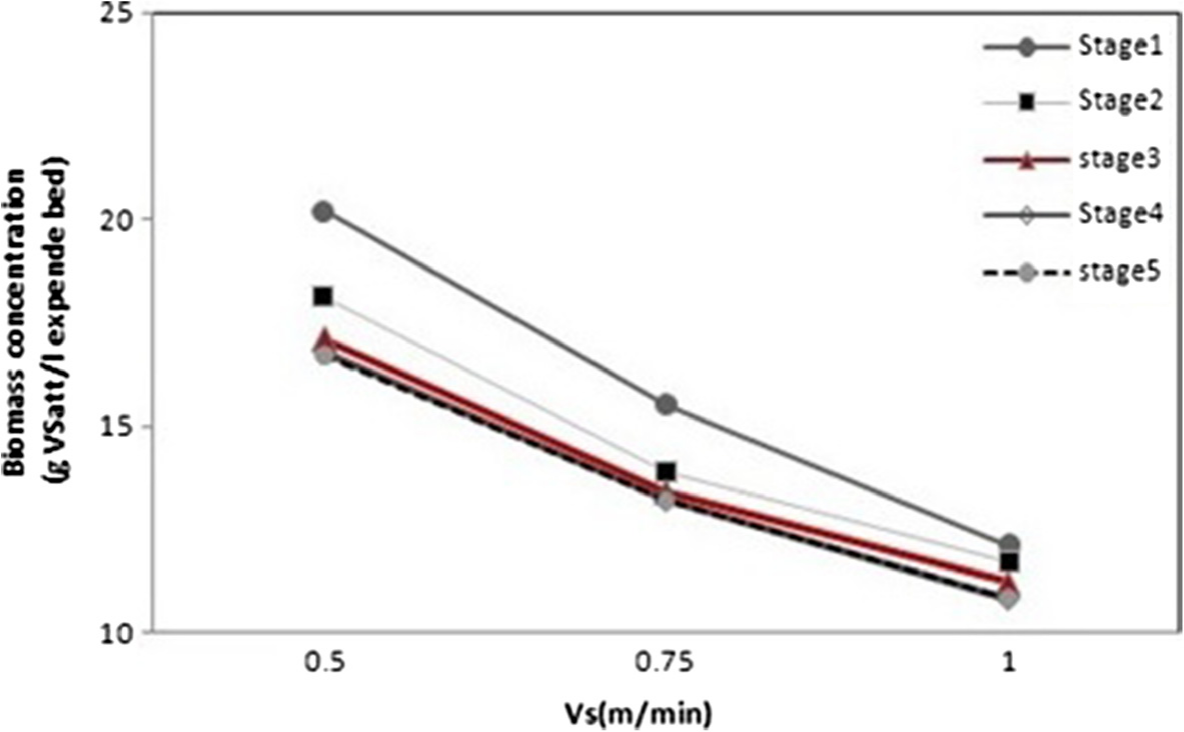
**Effect of upflow velocity in biomass concentration.**

**Figure 5:**
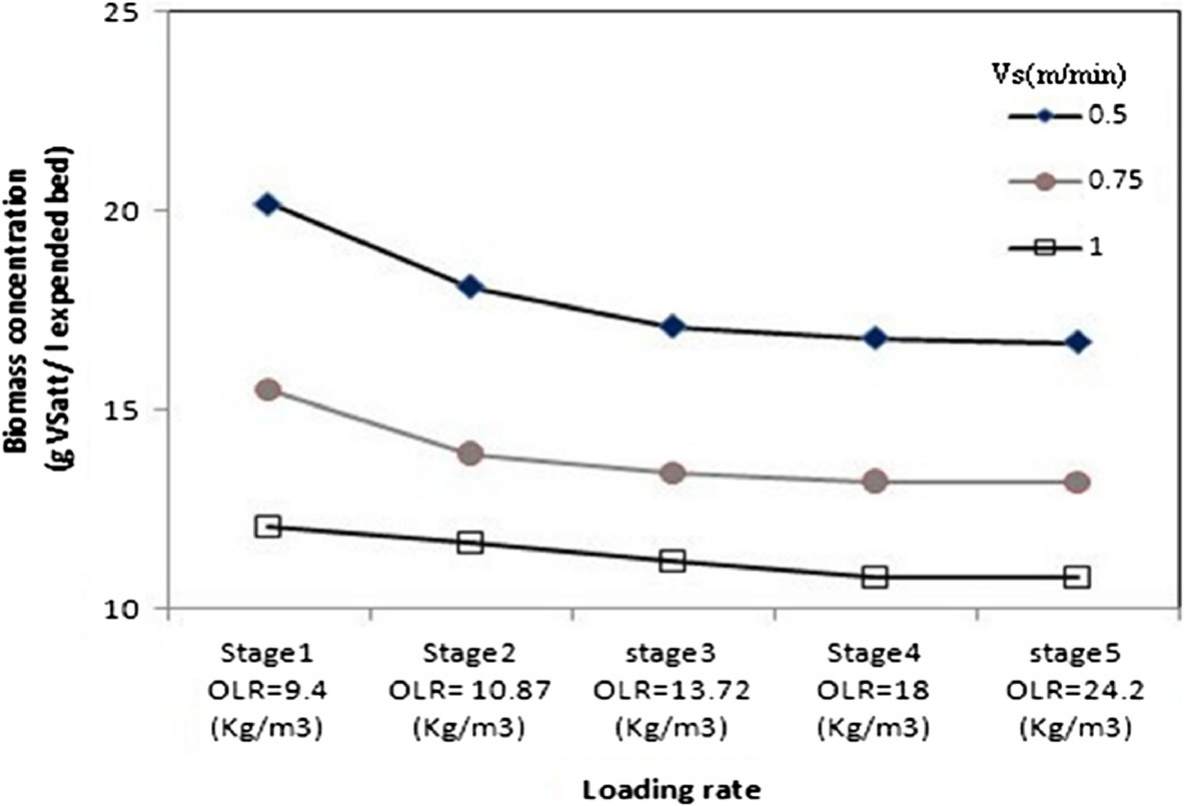
**Effect of organic loading rate in biomass concentration.**

The effect of the upflow velocity on the average biomass concentration in the AFBR is illustrated by Figure 4. As shown, that the average biomass concentration per unit volume of the AFBR decreased with the increase of the upflow velocity at all of organic loading rate. For example, at OLR of 9.4 g COD L.d^−1^ with HRT of 48 h, the average biomass concentration decreased from 20.2 to 12.1 g VSS L^−1^, when Vs was increased from 0.5 to 1 m min^−1^. The decrease in the average biomass concentration as a result of the increase in upflow velocity is attributed to two main factors. When, Vs was greater than before, the bed porosity increased, which resulted a lower concentration of bio-particles per unit volume of the AFBR and consequently a lower biomass concentration in volume of reactor. Furthermore, shear forces exerted on the biofilm by the fluid increased. This resulted in a denser and thinner biofilms were formed and consequently resulted in a lower biomass concentration. As shown in Figure 5, it was observed that the average biomass concentration in the AFBR generally decreased with increase in the organic loading rate up to stage 3, wherever the change of biomass concentration as a function of the organic loading rate became insignificant. As at Vs of 0.75 m min^−1^, the average biomass concentration decreased from 15.5 to 13.2 gvss L^−1^ expanded bed when the organic loading rate was increased from 9.4 to 10.87 g COD L.d^−1^, respectively. Then, when the loading rate was increased from 10.87 to 24.2 g COD L.d^−1^, the average biomass concentration accomplished an approximately steady value of 13.18 gvss L^−1^ expanded bed. Also, similar trend of results was obtained from the other sets of experiments. However, at Vs of 0.5 m min^−1^, the rate of change in the average biomass concentration started to decrease at a higher loading rate (13.72 g COD L.d^−1^) than that of the other two upflow velocities. The biomass concentration decreases when the organic loading rate increase occurs as a result of the increase in the biofilm thickness due to the increase in the substrate concentration in the bulk liquid [39]. Since, the biofilm thickness increased, the porosity of the AFBR rose, and therefore the average biomass concentration per unit volume of the bed decreased.

### Biomass concentration, biofilm thickness and particle density profiles along the AFBR

**Figure 6:**
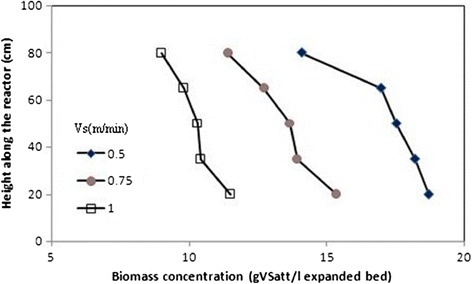
**Profiles of biomass concentration at different upflow velocities.**

**Figure 7:**
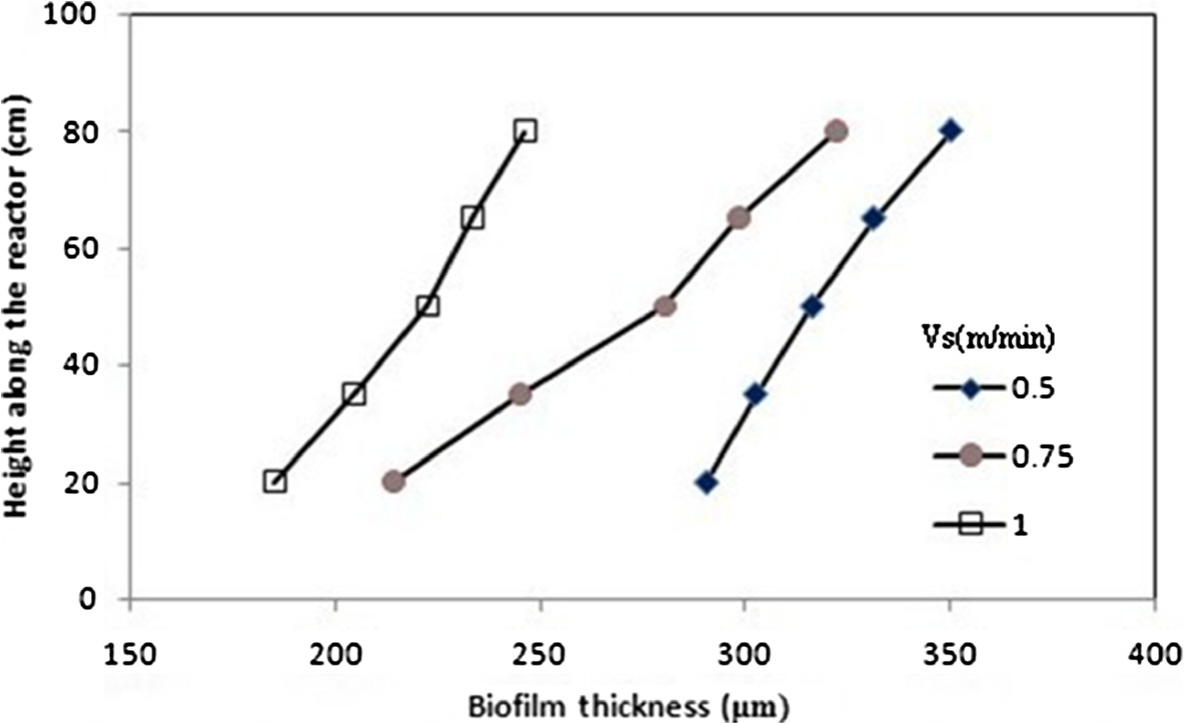
**Profiles of biofilm thickness at different upflow velocities.**

**Figure 8:**
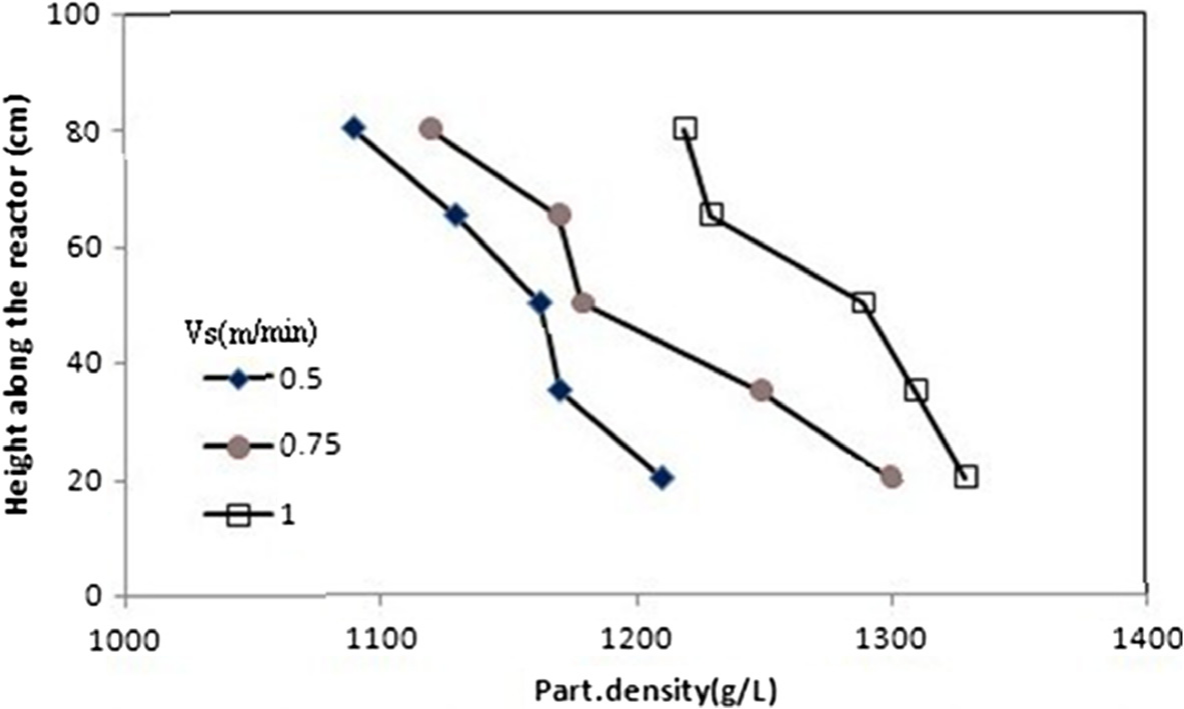
**Profiles of particle densiy at different upflow velocities.**

Figures 6, 7, and 8 show typical profiles of biomass concentration, biofilm thickness and particle density along the AFBR, respectively. As shown in Figure 6, as a result of the increase in the bed porosity along the reactor from the bottom to the top. In this figure, it was observed that biomass concentration as bio-particles per unit volume of the AFBR decreased along the reactor from the bottom to the top. As shown in Figure 7, biofilm thickness increased from the bottom to the top of the reactor representing a stratification of the bio-particle in the AFBR. Stratification is a consequence of the variability of the bio-particle densities in the reactor. Also, the unequal colonization of the substratum can be one of the causes of the variability of the bio-particle densities in the reactor.

Figure 8 shows the typical pattern of particle density along the AFBR. The biofilm created on the lower levels will be, probably, more dense than that formed in the upper levels as a result of the higher pressure exerted in this zone of the reactor, and this will create denser bio-particles. In the upper part of the bed, a biofilm with a lower density and, proportionally, greater thickness will develop because of the lower pressure presented in this zone. As reported by Zhang and Bishop, the biofilm densities differ with depth within the biofilm layers for the reason that the tops of biofilm are more porous as reported by Zhang and Bishop [40]. The densities in the top layers are usually 5–10 times higher than those in the top layers, and the porosities in the top layers are in the range of 84–93%, while it is in the range of 58–67% in the bottom layers [41]. The lower part of the reactor had denser bio-particles and consequently had lower bed porosity.

## Conclusions

Anaerobic fluidized bed reactor with particles made of PVC as the supporting material is highly effective for COD removal for high strength wastewater from currant wastewater. The results demonstrated that the AFBR system is capable of handling an exceptionally high organic loading rate with very high removal efficiency, up to 96.6%. The average biomass concentration per unit volume of the AFBR (as gVSSatt L^−1^ expended bed) decreased with increase in the upflow velocity at all the applied organic loading rates up to some loading rate as a result of the increase in the bed porosity. The total biomass in the reactor increased with increases in the organic loading rate. The peak biomass concentration (as gVSSatt L^−1^ expended bed) was observed at the bottom part of the reactor, then it droped off slowly towards the top. The biofilm thickness increased from the bottom to the top of the reactor representing a stratification of the media in the AFBR. The bed porosity increased from the bottom to the top of the reactor.
